# Recent Advances in Microfluidics-Based Monitoring of Waterborne Pathogens: From Isolation to Detection

**DOI:** 10.3390/mi16040462

**Published:** 2025-04-14

**Authors:** Guohao Xu, Gaozhe Cai, Lijuan Liang, Jianxin Cheng, Lujie Song, Rui Sun, Feng Shen, Bo Liu, Shilun Feng, Jin Zhang

**Affiliations:** 1 College of Life and Geographic Sciences, Kashi University, Kashi 844000, China; xuguohao3@163.com; 2 Jiaxing Key Laboratory of Biosemiconductors (A), Xiangfu Laboratory, Jiashan 314102, China; chengjianxin001@163.com (J.C.); rsunsinap@163.com (R.S.); shenfeng@tsinghua-zj.edu.cn (F.S.); liubo@xflab.org.cn (B.L.); 3 School of Microelectronics, Shanghai University, Shanghai 201800, China; caigaozhe@shu.edu.cn; 4 State Key Laboratory of Transducer Technology, Shanghai Institute of Microsystem and Information Technology, Chinese Academy of Sciences, Shanghai 200050, China; llj@mail.sim.ac.cn; 5 University of Chinese Academy of Sciences, Beijing 100049, China; 6 Department of Urology, Shanghai Sixth People’s Hospital Affiliated to Shanghai Jiao Tong University School of Medicine, 600 Yishan Road, Shanghai 200233, China; ljsong@sjtu.edu.cn; 7 College of Biological, Chemical Sciences and Engineering, Jiaxing University, Jiaxing 314001, China

**Keywords:** waterborne pathogens, microfluidic chip, isolation, detection, lab-on-a-chip (LOC), sample processing, nucleic acid analysis

## Abstract

Waterborne pathogens seriously threaten human life and can cause diarrhea, gastrointestinal disorders, and more serious systemic infections. These pathogens are usually caused by contaminated water sources that contain disease-causing microorganisms, such as bacteria, viruses, and parasites, which cause infection and disease when they enter the human body through drinking water or other means. Due to the wide range of transmission routes and the high potential risk of waterborne pathogens, there is an urgent need for an ultrasensitive, rapid, and specific pathogenic microorganism monitoring platform to meet the critical monitoring needs of some water bodies’ collection points daily monitoring needs. Microfluidics-based pathogen surveillance methods are an important stage towards automated detection through real-time and multi-targeted monitoring, thus enabling a comprehensive assessment of the risk of exposure to waterborne pathogens and even emerging microbial contaminants, and thus better protection of public health. Therefore, this paper reviews the latest research results on the isolation and detection of waterborne pathogens based on microfluidic methods. First, we introduce the traditional methods for isolation and detection of pathogens. Then, we compare some existing microfluidic pathogen isolation and detection methods and finally look forward to some future research directions and applications of microfluidic technology in waterborne pathogens monitoring.

## 1. Introduction

Water is one of the most important resources for the prosperity and security of the world. However, with global climate change and population growth, water supply and demand are gradually out of balance. Water pollution is one of the main causes of water scarcity. According to recent research data, by 2025, the number of affected basins and populations is expected to increase significantly. Hence, there is an urgent need to address water quality issues in water resource management projects [1]. Globally, there are unmanaged drinking water sources used by at least 2.2 billion people, and approximately 1.8 million people die each year due to contaminated water sources [2,3]. Waterborne pathogens are one of the most threatening sources of water pollution, and effective monitoring of waterborne pathogens is an important tool for the timely detection and management of water pollution [4,5].

Conventional methods for the detection of waterborne pathogens include culture, immunoassay, and molecular detection. Among these, culture remains the gold standard due to its high detection sensitivity. However, it requires a prolonged incubation step, typically extending detection time to 2–5 days [6]. Immunoassays offer rapid detection but suffer from low detection sensitivity and cannot distinguish between live and dead bacteria, which can easily lead to misjudgment [7]. The molecular detection method has high sensitivity and strong specificity but requires complex nucleic acid extraction steps and specialized laboratories, limiting their applicability for real-time on-site monitoring of pathogens [8,9,10]. Additionally, pathogens in natural water bodies are often present at low concentrations, yet they can spread rapidly [11,12]. Traditional detection methods face challenges in identifying pathogens from large volumes of water samples. To address this, enrichment techniques such as filtration, centrifugation, or adsorption are usually employed to increase the concentration of pathogens and thus enhance the sensitivity of detection [13,14,15,16].

Yuan et al. systematically summarized the core advantages of centrifugal microfluidics in prompt onset testing (POCT), including parallel sample processing, density-differential-driven valveless fluid control, and low-cost consumable design. However, its application scenarios are mainly for laboratory-grade samples such as blood, lacking targeted solutions for the pretreatment of complex water samples and relying on sophisticated temperature control equipment [17]. The review by Lee et al. focuses on the optimization of bacterial lysis techniques, comparing the advantages and disadvantages of chemical lysis, physical lysis, and enzymatic lysis, emphasizing the protective effect of physical lysis on the integrity of nucleic acids, and proposing magnetic bead purification coupled with microfluidic control for the removal of inhibitors from environmental samples. However, this technique requires offline lysis and purification steps, which makes it difficult to realize the integration of the whole process, and the residual inhibitors may interfere with the detection [18]. Liu et al. reviewed the application of microfluidics in the diagnosis of acute respiratory tract infections (ARTI), covering the pathogenesis and diagnostic challenges of pathogens such as pneumonia, influenza, SARS, and COVID-19. By integrating technologies such as nucleic acid amplification and antigen detection, the microfluidic system significantly reduces sample/reagent consumption and enhances detection throughput and speed with the advantages of miniaturization, automation, and high sensitivity. However, there is no mention of the technological development and application of utilizing microfluidics to isolate and detect waterborne pathogens [19].

With the rapid development of microfabrication technology, microfluidic chips have become a novel tool for monitoring waterborne pathogens [20,21,22]. Microfluidic chip-based methods combined with enzyme-linked immunosorbent assays (ELISA), polymerase chain reaction (PCR), surface-enhanced Raman spectroscopy (SERS), and other technologies have demonstrated exceptional performance in pathogen enrichment and detection. It can realize the automation of the entire process from sample preparation to detection and has the characteristics of short detection time, high throughput, and high sensitivity [20,23,24,25]. For instance, Byungrae et al. proposed a nanoplasmonic microfluidic chip for the preconcentration and lysis of *Escherichia coli* (*E. coli*) in less than 1 min and rapid identification combined with ultrafast photon PCR [26]. Zhao et al. developed a wax-printed paper-based ELISA for the detection of *E. coli* in 3 h with a detection limit of 10^4^ CFU/mL [27]. Microfluidic portability and ease of operation make them outstanding in resource-limited field environments, making them an important tool in the field of water quality monitoring [28].

**Figure 1:**
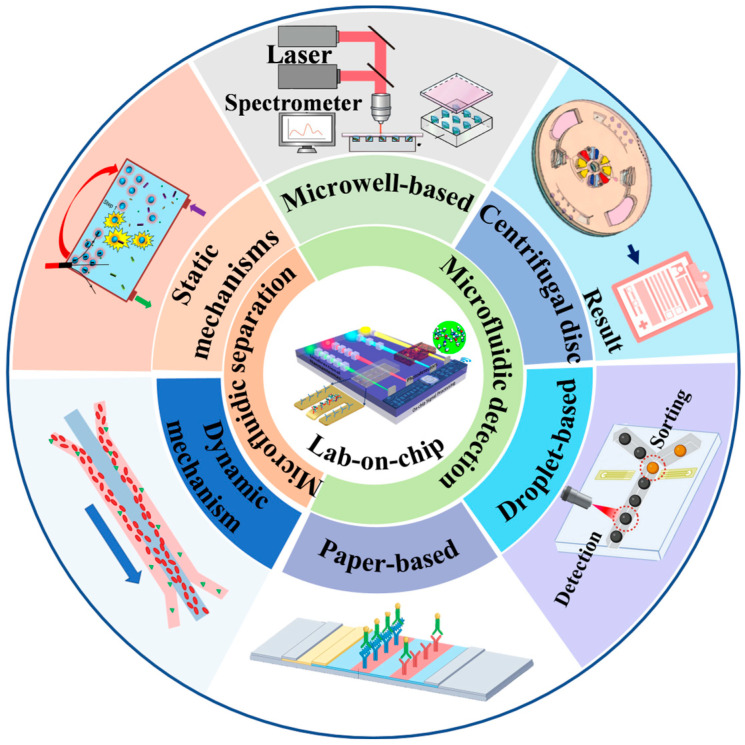
Microfluidics technology realizes efficient separation and highly sensitive detection of pathogens through integrated chip design (e.g., centrifugation, droplet encapsulation, paper-based detection, and other modules) and can be used in diverse application scenarios ranging from clinical diagnosis to environmental monitoring.

In this review, our aim is to present a thorough and in-depth overview of recent microfluidic methods for the monitoring of waterborne pathogens, along with some future research directions and applications. It will begin by summarizing traditional methods for waterborne pathogens enrichment and detection, assessing their respective merits and limitations. Then, we summarize some recent microfluidic methods for the separation of waterborne pathogens, such as membrane separation, electrical separation, and magnetic separation according to the external physical force. Meanwhile, the microfluidics-based detection methods of waterborne pathogens are also summarized. We review recent research on the application of microfluidics in the field of pathogen isolation and detection(Figure 1). Finally, the conclusion highlights some promising strategies to address the current shortcomings and challenges of real-time rapid detection.

## 2. Conventional Separation and Detection Methods

### 2.1. Conventional Separation Methods

Isolation of disease-associated microorganisms from complex biological mixtures and matrices is a key step in monitoring waterborne pathogens and can be categorized as physical or biochemical separation depending on the structure and properties of the pathogens.

**Figure 2:**
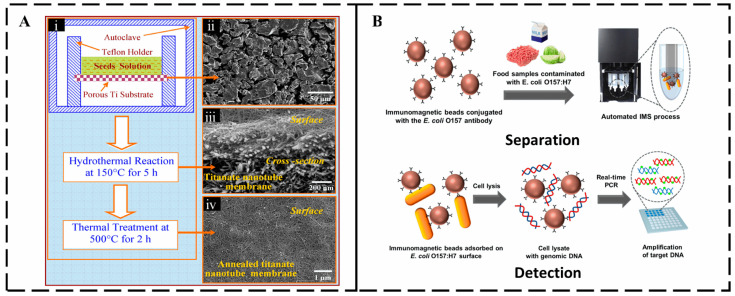
Conventional separation and detection methods (**A**) (**i**) Schematic illustrating a direct hydrothermal growth method for fabrication of TNM. (**ii**) Typical SEM image of the porous titanium membrane substrate. (**iii**) Typical SEM image of an as-synthesized TNM. (**iv**) Typical SEM image of an annealed TNM [13]. Copyright 2009, Elsevier. (**B**) Immunomagnetic separation (IMS) method for isolating and concentrating target bacteria from samples [31]. Copyright 2020, MDPI.

Physical separation methods are mainly based on the physical properties of pathogens, such as size, buoyancy magnitude, density, and charge difference. Traditional physical separation methods mainly include filtration and centrifugation. Filtration methods typically utilize filter membranes with specific pore sizes to effectively separate and purify pathogens from complex samples. As shown in Figure 2A, Zhang et al. successfully prepared a hierarchical titanium nanotube membrane (TNM) on porous membrane substrates by hydrothermal method, which can prevent cracks and pinholes from being generated, have high selectivity, high flux, and biocompatibility, and can effectively separate pathogens in water purification [13]. Madhukar et al. demonstrated that the removal of large particulate impurities by filtration is effective in reducing interference and retaining smaller microorganisms. In this work, bacteria-nanoparticle complexes were isolated using streptavidin-coated magnetic nanoparticles (MNCs), conjugated to biotin-labeled antibodies in the presence of a magnetic field with a capture efficiency of over 94%. This proposed method can successfully capture *E. coli* O157:H7 in samples with concentrations ranging from 1.6 × 10^1^ to 7.2 × 10^7^ CFU/mL in 15 min with a minimum capture efficiency of 94% [29]. Centrifugation is another important physical separation method, which usually includes differential centrifugation, density gradient centrifugation, ultracentrifugation, and isodensity centrifugation. Liu adjusted the centrifugal force to 6000× *g*, enabling effective separation of *E. coli* from fecal liquid samples, with minimal impact on bacterial enrichment [30]. Park et al. used automated immunomagnetic bead separation (IMS) technology to achieve efficient capture and enrichment of more than 99% of *E. coli* O157:H7 from pre-enriched milk samples in less than 10 min using magnetic beads modified with target-specific antibodies, significantly improving detection sensitivity. When combined with an enzymatic colorimetric reaction, the system can detect pathogens as low as 3 × 10^2^ CFU/mL within 3 h with 100% specificity, effectively avoiding cross-interference (Figure 2B) [31].

Despite the high efficiency of physical methods in isolating pathogens, there are still significant limitations for rapid detection in the field: first, lack of specificity; it is difficult to distinguish pathogens from non-targeted impurities (dead bacteria, debris, or similar particles) based on physical attributes such as size, density, etc., and the efficiency of capturing samples of low concentration (<10^1^ CFU/mL) is reduced dramatically; second, the risk of cellular damage is high, and the structural integrity of the pathogen is easily damaged by high-speed centrifugation (6000× *g*) or mechanical filtration, resulting in leakage of intracellular material and decreased activity, leading to the leakage of intracellular substances and the decline of activity, which affects the subsequent culture or molecular detection; third, the application efficiency is limited. Complex samples (high viscosity/multiple impurities) can easily lead to the clogging of membranes or centrifugation and delamination difficulties, and the large-scale application of the application is limited by the high cost of filtration membranes (titanium nanomembranes), the dependence on the equipment, and the difficulty of standardization of the operation. It is also unable to achieve efficient enrichment and biorecognition at the same time and needs to rely on auxiliary technologies such as antibody labeling to improve selectivity. These shortcomings limit its usefulness in precision diagnosis and rapid on-site detection.

Biochemical isolation methods mainly leverage molecular interactions, enzymatic reactions, affinity differences in growth habits, and other chemical properties specific to different pathogens. These methods mainly include plate cultivation, precipitation, affinity chromatography, ion exchange chromatography, and solvent extraction. Among these, the plate method has long been used for microbial isolation and remains a fundamental biological technique. However, it is time-consuming, requires a fully equipped laboratory, and relies on subsequent identification steps. Sedimentation is an isolation technique in which pathogens are precipitated from a solution by adding salts, metal ions, or organic solvents to the pathogen sample. Nair et al. achieved rapid, specific, and highly sensitive isolation of *Mycobacterium tuberculosis* by utilizing the ability of the C-terminal domain (CTD) to specifically bind *Mycobacterium tuberculosis* peptidoglycan (PG) by magnetic bead adsorption. This method demonstrated 100% sensitivity and specificity in isolating *Mycobacterium tuberculosis* [32]. While this approach effectively addresses issues of poor selectivity, it remains time-consuming and is limited in terms of precision, efficiency, and control. Schaumburg et al. further utilized immunomagnetic adsorption with antibody-modified magnetic particles and free antibody conjugated to β-galactosidase (β-gal) to isolate and pre-concentrate *E. coli* by a magnetic field, reducing the enrichment time to less than half an hour, and enriching the concentration from 9.2 CFU/mL to 9.2 × 10^7^ CFU/mL [33]. The shortcomings of biochemical isolation methods are centered on complex and time-consuming procedures, such as multiple incubation and washing steps for immunomagnetic beads, which cost more than USD 20 per incubation and rely on expensive specialized equipment. Interference of serum components in complex samples significantly reduces the capture rate, and the capture efficiency of low-abundance pathogens exponentially decreases with decreasing concentration. Chemical reagents may damage the structure of pathogens, affecting subsequent activity analysis. In addition, the stability of biomolecules is susceptible to environmental fluctuations, poor batch-to-batch reproducibility during process scale-up, and high standardization difficulties.

Traditional methods for the separation and enrichment of waterborne pathogens have provided a solid foundation for effective detection, but they still face some challenges. Filtration is a simple and cost-effective technique, well-suited for removing larger impurities. However, its selectivity for pathogens remains limited. Centrifugation, on the other hand, offers high resolution and accurate separation based on density differences, making it effective for isolating microorganisms. Despite these advantages, the high cost of centrifugation equipment and the complexity of the operation hinder its large-scale application.

### 2.2. Conventional Detection Methods

Microbiological laboratory methods for detection are mainly based on purification isolation and culture, which are the main methods for pathogenic bacteria detection due to their sensitivity and reliability, including pour plate, smear, line, and serial dilution methods [34,35]. The use of yeast-extract agar-decantation plates is a standard method for the detection of common heterotrophic bacteria in drinking water, allowing for the counting of maximum probable numbers of heterotrophic bacteria in drinking water, including *E. coli* (O157: H7, O26, O103, O111, and O145) and *Salmonella*, with a limit of detection as low as 1.0 log CFU/100 mL [36,37]. Sourav et al. were able to selectively detect *Vibrio cholerae* with 100% specificity and sensitivity by adding different combinations of sodium chloride (10~13%) and ferrous sulfate (8 mM~15 mM) to yeast peptone dextrose (YPD) agar and incubating the agar for 2~3 days at 37 °C, 40 °C and 42 °C, respectively [38]. However, in this method, the steps of culture, isolation, and purification are time-consuming and require professional operation, which makes it difficult to accomplish the goal of real-time on-site monitoring.

Molecular methods identify and quantify pathogens through the use of molecular markers such as nucleic acids or proteins, mainly including nucleic acid amplification, probe hybridization, and gene sequencing, which can greatly reduce the detection time [39,40]. Molecular methods can quickly and accurately detect and identify *Cryptosporidium parvum* and *Cyclospora cayetanensis* and are widely used in environmental monitoring and diagnosis of waterborne parasites. Sampling operation and detection times take more than 5 h [41,42]. Montserrat et al. developed a peptide nucleic acid fluorescence in situ hybridization (PNA-FISH)-based method using the LEG22 PNA probe that can specifically detect *Legionella* species with a specificity of 99.9% and a sensitivity of 96.9% and showed a good signal-to-noise ratio in artificially contaminated water samples. However, for highly turbid samples, pretreatment is required to overcome autofluorescence interference and achieve continuous detection, shortening the detection time to less than 3–5 h [25]. The simple and rapid syringe filter-based DNA extraction method and portable detection platform developed by Seunguk et al., combined with loop-mediated isothermal amplification (LAMP) technology, can detect *E. coli* in water within 30 min [10]. The method provides an effective approach for on-site testing environments lacking laboratory equipment and technicians and lays the experimental foundation for subsequent microfluidic testing.

A variety of immunological methods have been developed for pathogen detection for more than 40 years, including ELISA, immunofluorescence assay (IFA), Western blot, colloidal gold immunochromatography, and flow cytometry, which provide new options for pathogen detection. ELISA uses the specific binding of antigen-antibody and the detectable signals generated by the enzymatic reaction to identify and quantify pathogens. As early as 1994, Tayler had already used ELISA to detect four bacteria simultaneously [43]. Xue et al. achieved sensitive detection by improving the ELISA method by immobilizing *Staphylococcus aureus* captured by vancomycin and butyrylcholinesterase-functionalized magnetic beads in a 96-well plate with the addition of gold nanoparticles and substrate, which catalytically generates a product that turns from red to purple [44]. Bayat et al. have designed two IgY-based sandwich ELISA reagents that provide 100% specificity for the detection and identification of toxigenic *Vibrio cholerae* [9]. ELISA is more sensitive than current rapid tests, making it a reliable and robust mass screening tool.

Traditional isolation and detection methods provide stable samples but have significant limitations in terms of efficiency, cost, and ease of operation: purification cultures take days and are only suitable for taxonomic identification; and nucleic acid hybridization (PCR/ELISA) is highly sensitive, but relies on costly equipment and complex operation, making it difficult to meet the needs of field trials. In contrast, microfluidic systems achieve technological breakthroughs through miniaturization and integrated design: their nanoscale fluidic manipulation reduces sample/reagent consumption by more than 90%, and the integrated chip integrates sample pretreatment, reaction, and assay into a single process, which shortens the traditional hours of reaction to minutes and avoids multi-step operation errors. In addition, the compatibility of microfluidic technology with molecular tools such as CRISPR enables single-copy sensitivity detection in complex samples, effectively solving the core problems of impurity sensitivity and high false-negative rate of traditional methods, providing a revolutionary solution for rapid on-site detection of pathogens.

## 3. Microfluidics-Based Separation

For water sample analysis, pathogens exist in complex matrices, and conventional separation and detection rely on different equipment and consumables. Through its miniaturization, integration, and automation features, microfluidic technology effectively overcomes the limitations of traditional waterborne pathogens separation methods in terms of efficiency, sensitivity, cost, and portability. With advances in materials science, manufacturing processes, and artificial intelligence, microfluidic systems are moving from laboratory research to practical applications such as clinical diagnosis and environmental monitoring. Particularly in resource-limited areas, emergency response and daily monitoring, microfluidic technology shows advantages that cannot be matched by traditional methods. In this section, the principles, applications, and development prospects of on-chip pre-enrichment technology for waterborne pathogens in the past decade will be discussed according to static and dynamic methods.

### 3.1. Static Separation Mechanisms

Static separation is a microfluidic structure without obvious liquid flow, using the pathogen’s characteristics, such as surface charge, antibody-antigen interaction, and hydrophilicity. The main components include filtration structure, electric field separation, and magnetic capture. Static separation can effectively remove substances in blood, saliva, and urine samples that interfere with the detection and analysis of pathogens, isolate pathogens, and reduce pretreatment time.

**Figure 3:**
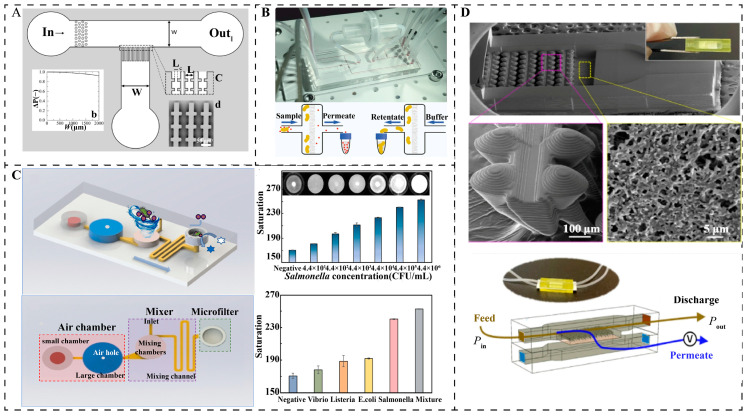
Static mechanisms (**A**) Overview of the multiplexed cross-flow filtration micromode [48]. Copyright 2018, Springer Nature. (**B**) An integrated membrane valve microfluidic platform for ultrafiltration control [49]. Copyright 2022, Springer Nature. (**C**) Schematic diagram of a microfluidic detection chip based on finger-driven mixing and core-track membrane filtration for rapid and sensitive detection of *Salmonella* [50]. Copyright 2023, Elsevier. (**D**) Bio-inspired anti-fouling membrane filtration device enabled by 3D printing-on-membrane [51]. Copyright 2022, Springer Nature.

In microfluidic systems, filtration structures were one of the first technologies to enable efficient, flexible, and controllable pathogen capture [45,46,47]. Membranes are porous or dense barriers for the selective passage of certain mixtures in fluids and are the key building blocks of filtration structures, which can be classified according to their structure as embedded membrane filtration chips and analog membrane filtration chips, as shown in Figure 3A [48]. Embedded devices are a method that combines traditional filtration with microfluidics. The membrane is housed in the microfluidic device to achieve effective separation. Ryzhkov et al. used multilayer soft lithography and biocompatible materials to fabricate an embedded microfiltration structure that integrates six pressure-driven membrane valves for automated, high-throughput *E. coli* separation and preconcentration, such as that illustrated in Figure 3B. The device achieved a separation efficiency of 81.33% in a 1 mL *E. coli* sample with a throughput of 120.42 μL/min, but the chip relied on a pressure-pumping device [49]. Jin et al. combined immune gold@platinum nanoparticle labeling technology with microfiltration technology to develop a finger-driven microfluidic chip for rapid enrichment and detection of *Salmonella typhimurium* without the need for external equipment such as that shown in Figure 3C. The finger-driven air chamber promotes the thorough mixing of the sample and nanoparticles, and the microfilter is used to capture bacterial conjugates, achieving enrichment within 25 min. The technology is simple and low-cost, but the microporous membrane is expensive and has poor selectivity for pathogens of similar size [50].

To address the shortcomings of traditional embedded membranes, researchers have begun to design microfluidic chips that mimic membrane structures, replacing the sorting role of pores in real membranes with parallel channels or microcolumns. Li et al. have designed self-sealing “gill”-structured membrane-on-a-chip devices for wastewater filtration by micro stereolithography 3D printing that can be easily embedded in metallic micromesh and polymer membranes without additional assembly. The biomimetic device uses hydrodynamic operations to efficiently eject dirt and avoid clogging and provides two to three times the filtration durability of commercial membrane devices when treating wastewater with plastic particles and emulsified oil droplets such as that shown in Figure 3D. This approach successfully combines microfluidics and membrane technology to enhance high-throughput filtration performance for applications in energy, sensing, and water treatment [51]. The simulated membrane device enables the observation of the individual behavior of particles and facilitates the observation of multilayer structures and pore blockage mechanisms through lateral two-dimensional observation.

Static separations utilize pathogen characteristics to achieve efficient separations in microfluidic systems through filtration, electric field, and magnetic capture. Embedded and analog membrane structures have improved separation efficiency by combining traditional filtration techniques and innovative designs, respectively, but suffer from cost or selectivity issues. Recent analog membrane structures have significantly improved high-throughput filtration performance for a wide range of applications, including water treatment and sensing.

### 3.2. Dynamic Separation Mechanism

The principle of microfluidic dynamic separation of pathogens is mainly to use physical force fields (such as optical, electrical, inertial, or acoustic) based on the specific physical or biological properties of pathogens (such as size, shape, charge or surface properties) to achieve rapid and effective separation and enrichment, and for clinical diagnosis and detection applications [52,53,54].

#### 3.2.1. Optical Separation Methods

**Figure 4:**
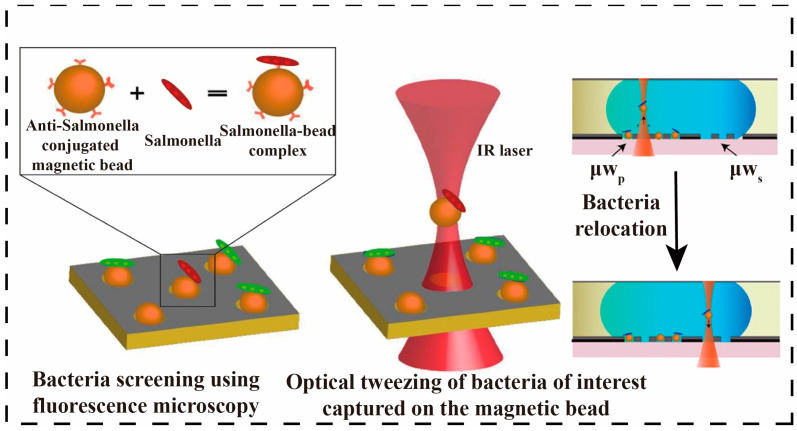
Optical separation. *Salmonella* bacteria bind to MBs conjugated with anti*Salmonella* antibody, thereby forming the *Salmonella*–bead complex, which is subsequently captured using infrared (IR) laser-based OT [57]. Copyright 2020, MDPI.

Microfluidic optical separation is a method of precisely controlling and separating tiny particles or cells using a combination of microfluidics and optics. Combining infrared optical fiber, Raman scattering, and fluorescence spectroscopy with techniques such as optical coherence tomography, interference, polarization, and laser spot, the manipulation of photon wavelengths and intensities in a microfluidic structure allows for the capture, manipulation, and separation of different pathogens in mixed samples [55,56]. Integration of digital microfluidics (DMF) with optical tweezers (OT) enables selective capture, transfer, and culture of individual bacteria. Fluorescently labeled *Salmonella typhimurium* was captured on the DMF platform by antibody-functionalized magnetic beads, followed by selective capture and transfer of these complexes using OT, which is shown in Figure 4. The method enables spatial organization and selection of bacteria in a two-dimensional format with single-cell resolution [57]. Separation of pathogens using nano photo sensors could further enable high sensitivity and portability [58]. Liu et al. further integrated optical through optical forces. The chip uses optical gradient force to capture *E. coli* while pushing red blood cells along the light propagation direction by optical scattering force. Experimental results showed that the chip successfully captured *E. coli* and pushed red blood cells in a mixed blood solution. The effect of the fiber tip angle on the cell potential for efficient cell and bacterial separation at low optical power [59]. Although flow-controlled optical separations are highly efficient and precise, their high equipment cost, technical complexity, clogging, and sensitivity to sample concentration limit their widespread use.

#### 3.2.2. Electrical Separation Methods

**Figure 5:**
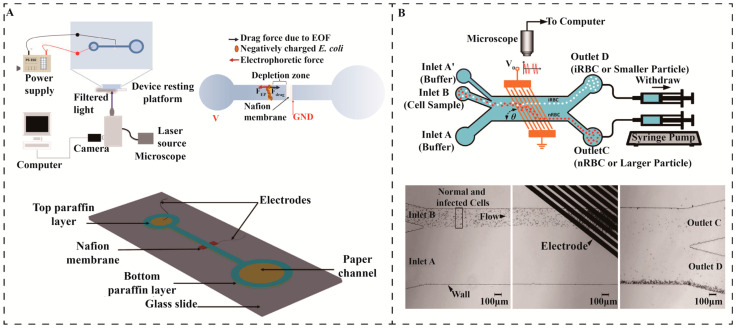
Electrical separation. (**A**) A schematic of the experimental setup used for concentration of *E. coli* by ICP experiments [63]. Copyright 2020, Wiley. (**B**) A discrete dielectrophoresis device for the separation of malaria-infected cells [64]. Copyright 2022, Wiley.

The electric field uses the net negative charge and membrane potential generated by the charged functional groups on the bacterial cell membrane to adjust the strength and direction of the electric field, dynamically control and separate specific types of pathogens, mainly relying on ion concentration polarization (ICP), microchip capillary electrophoresis (MCE) and dielectrophoresis (DEP) [60,61,62]. Perera et al. designed a microfluidic paper-based separation device for the rapid preconcentration of *E. coli* in water using ICP. The device consists of a paper channel with a nafion^®^ membrane and a microfilament electrode, which induces the ICP effect by voltage. The fluorescence method of SYTO 9 staining was used for the detection of *E. coli*, and the device achieved up to a concentration factor of 2 × 10^5^ times in a few minutes, demonstrating its high efficiency for the detection of *E. coli* in high salinity and freshwater environments such as that shown in Figure 5A. The device is simple to manufacture, low-cost, disposable, and suitable for rapid water quality monitoring [63]. However, due to poor biocompatibility, complex electric field regulation, limited applicability, and sample matrix affecting the separation efficiency, the application of ICP in pathogen separation is relatively rare.

DEP has become one of the effective techniques for the mechanical separation of pathogens due to its simple structure and no cell damage [62]. Radin et al. used embedded nanopore sensors to optimize the electric field distribution near the nanopore using a pair of electrodes in a circular flow channel to generate a symmetrical electric field, thereby achieving precise separation of double-stranded DNA [45]. Kamuri et al. proposed a mechanism for separating microorganisms using pulsed-field dielectrophoresis field flow separation (DEP-FFF). By changing the pulse duration and flow rate while keeping the frequency, voltage, and conductivity constant during the separation process, yeast and *E. coli* were best separated at a longer pulse duration (12 s) and a faster flow rate (9.6 μL min^−1^) [54]. Panklang et al. combined the discrete DEP technique in a microfluidic device by modulating the magnitude, frequency, and duty cycle of the voltage to apply DEP force to deflect normal erythrocytes and separate them from malaria-infected cells [64].

Positive DEP (pDEP) was utilized to move the normal erythrocytes away from the target area while the infected cells remained in place to avoid channel blockage, such as that shown in Figure 5B. Experimental results show that the enrichment of infected cells is 3000 times higher at the output of the device than at the input, demonstrating that this technique is effective in separating and detecting malaria-infected cells in highly concentrated cell samples and that it is suitable for high-throughput isolation under conditions of hypoparasitemia [64]. Although DEP can effectively separate pathogens, its electrical properties have limited selectivity, the device structure is complex, and it is highly dependent on electric field parameters. It is prone to clogging when processing high-concentration samples and may produce thermal effects and cell damage, which limits its application in large-scale, high-throughput applications.

#### 3.2.3. Fluidic Separation Methods

Fluidic separation is mainly based on the fluid forces generated by its microscale geometry, including deterministic lateral displacement (DLD), inertial force, and hydrodynamics. It is necessary to rely on the size difference between the target bacteria and other particles to separate the target bacteria from the sample without any external energy participation. It can effectively separate micron-sized target bacteria and is suitable for separating pathogens in water samples.

DLD consists of flattened microfluidic channels filled with a regular series of microcolumn obstacles and has been successfully used for the isolation of yeast, spores, bacteria, viruses, and DNA [65,66]. Pariset et al. designed a device capable of successfully extracting *E. coli* from prostate cancer-containing blood samples by connecting multiple DLD devices in series and combining them with the use of flexible chambers for continuous sample collection and injection, achieving 100% removal of cancer cells and 93% removal of erythrocytes [67]. To simplify the DLD information processing process, Gioe et al. built an automated tool for particle flow observation and characterization in high-throughput experimental DLD devices by combining probabilistic Hough transform, canny edge detection, and Python template matching. The tool combines automatic frame flipping and template matching with machine vision to achieve an overall particle detection accuracy of 97.86% in an average computation time of 25.274 s, significantly reducing analysis time [65]. Wunsch et al. further reduced the gap of the DLD array to 25 nm and separated colloids as small as 20 nm at Pe ≥ 4, exceeding the efficiency limitations of the microscopic model [68].

**Figure 6:**
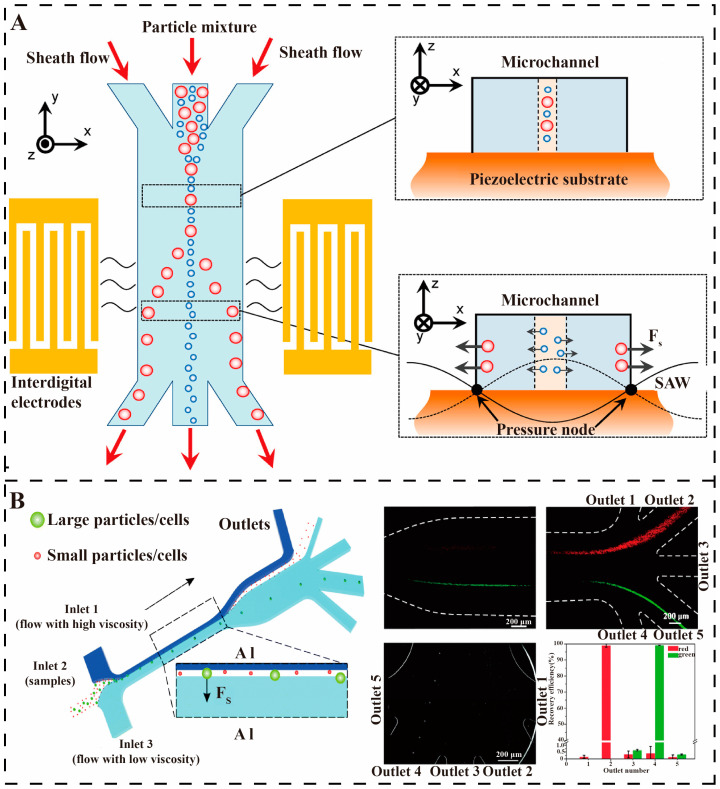
Fluidic separation. (**A**) Schematic illustration of the microfluidic device and separation mechanism using a SSAW field [69]. Copyright 2013, American Chemical Society (**B**) Separation of bacterivorous jakobid flagellate by sharp velocity gradient-induced soft inertial forces [70]. Copyright 2018, Royal Society of Chemistry.

Inertial microfluidics has a significant impact on high-throughput separation applications in environmental purification and physiological fluid handling, as well as bioparticle-focusing applications in clinical diagnostics, whereas its performance is mainly affected by the channel wall, channel curvature or perturbation structure, particles, and media properties [21,71]. Seo first introduced inertial microfluidics in 2007 for continuous, low-cost, and high-throughput separation, designing particle size cutoffs in the channel to concentrate and divert the particulate stream [72]. Ganz et al. further developed a microfluidic chip based on inertial separation principles, comprising a main separation channel (SC) and three exit channels, to efficiently separate encapsulated *Giardia flagellaris* from impurities. The sample was introduced into the chip via a syringe pump, allowing smaller impurities to be discharged into the waste stream while retaining and concentrating the encapsulated *Giardia flagellaris*. To enhance separation and visualization, the main separation channel was expanded into a larger channel (LC) to reduce the flow rate. The results demonstrated that the chip successfully removed 70% of impurities and concentrated 82% of the encapsulated *Giardia flagellaris*, significantly reducing background interference and analysis time [73]. In 2018, Pan et al., on the other hand, successfully removed more than 94% of live bacteria using a soft inertial separation chip with a low-speed, high-viscosity sheath flow and a high-speed, low-viscosity sheath flow in a cell stream (as shown in Figure 6B). The separation efficiency of the chip increased by more than 40% compared to the previous technique [70]. Nam et al. proposed a microfluidic chip based on hydrodynamics, in which the separation, mixing, and concentration of bacteria can be achieved by dean flow and inertial focusing. The microchip utilizes a spiral-shaped fluidic channel so that particles of different sizes are subjected to different centrifugal forces in the fluid to achieve the separation of *E. coli*. The separation efficiency of *E. coli* from 4.1 μm particles was 87.07 ± 2.21%. The real-time separation capability of the chip demonstrates its potential for use in disease diagnosis [74]. However, challenges remain, with larger pathogens tending to stay on the inner side of the spiral channel and smaller particles moving toward the outer wall. Further research is required to predict the focusing mass and optimize the design of inertial microfluidics.

Hydrodynamics is the power source and fundamental technology for the controlled manipulation and separation of pathogens. Amit utilized multiple hydrodynamic effects in a network of microfluidic channels for the separation of bacteria and suspended impurities in water. The results show an average efficiency of 99.6% in separating suspended particles and microorganisms in the 1–10 micron range [75]. The combination of acoustic and piezoelectric technologies and microfluidics has demonstrated significant advantages in the field of waterborne pathogen separation, where non-contact, high-precision separation of pathogens can be achieved by surface acoustic waves (SAW) [76]. For example, Ai et al. developed a microfluidic separation device based on standing surface acoustic wave (SSAW), which used a 128° YX-cut lithium niobate substrate to fabricate a fork-finger transducer (IDT), generating an acoustic field with a wavelength of 200 μm at a frequency of 20 MHz, and realizing the particle-size-dependent separation by acoustic radiation force. The experiments were performed using a dual-sheath flow focusing technique to compress the sample flow to a narrow band of 20 μm. Under 371 mW input power, 1–2 μm *E. coli* and 7–12 μm peripheral blood mononuclear cells (PBMCs) were subjected to acoustic radiative forces of 0.5 and 15 pN, respectively, which resulted in a displacement difference of 150 μm and the final purity of the separations reached 95.65% and 91%. This technique optimizes the acoustic-fluid coupling parameters through COMSOL multiphysics field simulation, uses a pulsed acoustic field (30% duty cycle) to control the temperature rise < 2 K, and combines with PEG modification on the PDMS surface to achieve a bacterial survival rate > 98% (Figure 6A) [69]. However, this technique also has the limitations of 30% energy loss due to PDMS acoustic impedance mismatch, the need to improve the resolution of pathogens of similar sizes and the possibility of high turbidity water samples interfering with the acoustic wave propagation; piezoelectric technology needs to solve the problems of material fatigue and cost, but the research and development of new biodegradable molecular ferroelectrics provide a material breakthrough for portable devices.

Modern microfluidic technology has made significant advances in pathogen detection. Optical separation technology, which combines laser focusing with microfluidic systems, enables precise control and separation of pathogens in samples. Techniques such as isoelectric separation via ICP and DEP further enhance bacterial separation efficiency by adjusting electric field parameters. Additionally, inertial microfluidics increases separation efficiency through the design of channels with particle size cutoffs and hydrodynamic focusing, providing a more effective and accurate solution for pathogen monitoring.

## 4. Microfluidics-Based Detection

### 4.1. Microwell-Based Detection of Waterborne Pathogens

Among various microfluidic platforms, microwell has the advantages of simple operation, easy integration, and strong in situ analysis capability, and has been widely used for the analysis of nucleic acids, proteins, and small molecules, which is one of the effective platforms for performing single-cell detection [77]. A size-selective microtiter plate fixation method, together with fluorescence microscope observation and a portable optical detection system, was used to establish a platform for the qualitative and quantitative detection of waterborne pathogens [57]. We will present the design and preparation of microtiter chips, including shape design and material selection, and discuss target and microtiter chip-based pathogen lysis strategies and detection methods.

**Figure 7:**
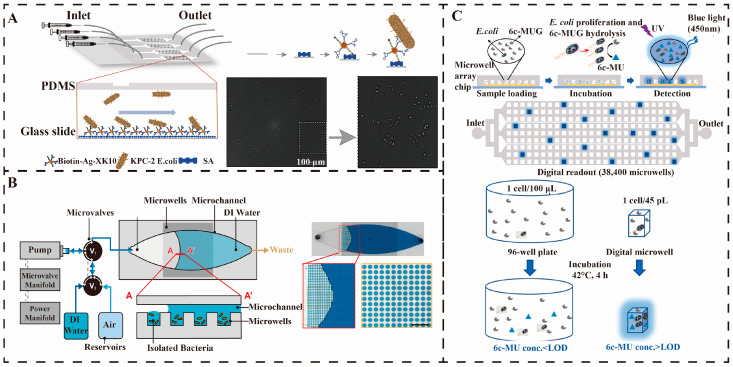
Microwell-based detection of waterborne pathogens. (**A**) Schematic drawing of the KPC-2 *E. coli* detection system [78]. Copyright 2020, Elsevier. (**B**) Microfluidic device for detecting *E. coli* using Raman scattering technology [79]. Copyright 2020, Elsevier. (**C**) Real-time monitoring and surface domain-based fluorescence analysis of single *E. coli* EK-19 detection in a microporous array [80]. Copyright 2022, Elsevier.

Microtiter platforms for the detection of different bacteria can be adapted to the detection needs of various microenvironments and can reveal cellular heterogeneity in greater depth. Chen et al. designed a microfluidic chip with integrated aptamer-modified Ag10NPs nano biosensors that can be combined with brightfield imaging for the rapid detection of klebsiella pneumoniae carbapenemase-2(KPC-2) expressing *E. coli*. The specific binding of the aptamer to the target bacteria allows direct observation and counting of the captured bacteria by bright field imaging. The detection process involves introducing the bacterial sample into the microfluidic chip at a flow rate of 30 mL/h, and after a 60-min reaction, it is washed to remove unbound material. The results showed that the microchip had a capture efficiency of more than 90% for KPC-2 *E. coli* with a detection limit of 10^2^ CFU and a detection time of approximately 1 h, as shown in Figure 7A. This system demonstrates the potential for rapid and specific detection of antibiotic-resistant bacteria but is currently only applicable to *E. coli* [78]. Huang et al. developed a microtiter-SERS assay chip, which enables in situ antibiotic susceptibility testing (AST) by encapsulating bacteria in microtiter wells in combination with SERS technology. The system is simplified into a two-layer structure for easy Raman microscopy scanning, and the centrifugation and air-drying process effectively enriches low concentrations of bacteria (10^3^ CFU/mL). The chip can shorten the bacterial incubation time by increasing the bacterial concentration, enhancing the strength and homogeneity of the SERS signal, and utilizing the porous structure to enable multiple parallel AST treatments, such as that shown in Figure 7B. The AST results for *E. coli* and *S. aureus* within 2 h were in agreement with the standard method, validating its reproducibility. However, the uneven distribution of bacteria at low concentrations remains a key issue for the system to be optimized [79]. As illustrated in Figure 7C, a chemiluminescent microarray chip for the rapid detection of individual active *E. coli* using a single bacterial immobilization structure developed by Wu et al. In the microarray chip, individual bacteria can be encapsulated in the microwells, and β-D-glucuronidase (GUS) can hydrolyze 6-chloro-4-methylumbelliferyl-β-D-glucuronide (6c-MUG) to release fluorescent molecules through GUS hydrolysis, which can be observed under a low magnification microscope. The method not only allows differentiation of different strains of *E. coli*, but the detection limit reaches the level of a single bacterium [80]. The versatility and flexibility of microtiter microarrays provide the advantage of loading samples without a pump and capturing and detecting secretions on glass slides. Microarrays can integrate single-cell culture and in situ analysis, helping to understand single-pathogen characteristics and phenotypic changes and provide insight into the molecular basis of bacterial function. However, the chip size limits the overall throughput capacity, the precision of environmental control is low, and cross-contamination between microwells remains a challenge. Nevertheless, microtiter technology still has the potential for capturing and detecting multiple targets in single cells. In the future, microwell-based waterborne pathogens detection technology will develop towards high throughput, intelligence, and multifunctional integration. By combining microfluidic chips with artificial intelligence algorithms, such as machine learning-optimized signal analysis, the system enables rapid identification of pathogens at low concentrations in complex water samples. In addition, the introduction of novel materials will enhance capture efficiency and signal sensitivity, while portable designs promote real-time monitoring in the field. Multi-omics coupling is expected to solve the challenges of drug-resistant gene detection, and ultimately form an integrated solution from the sampling to the analysis, serving the environment and public health safety.

### 4.2. Centrifugal Microfluidic Detection of Waterborne Pathogens

The simple, robust, and controllable operating principles of centrifugal microfluidics give it advantages in the analysis and immediate diagnosis of waterborne pathogens, such as reduced instrumentation use, effective bubble removal, and density-based sample separation. Most fluid handling functions can be realized by frequency-controlled interactions between inertia (centrifugal, Eulerian, and Coriolis forces), capillarity (hydrophilic/hydrophobic), and fluid structure on the micrometer-to-millimeter scale. This section focuses on the integrated implementation of waterborne pathogens unit operations and complex analytical assays in centrifugal microfluidics and looks at the potential and challenges of the technology.

**Figure 8:**
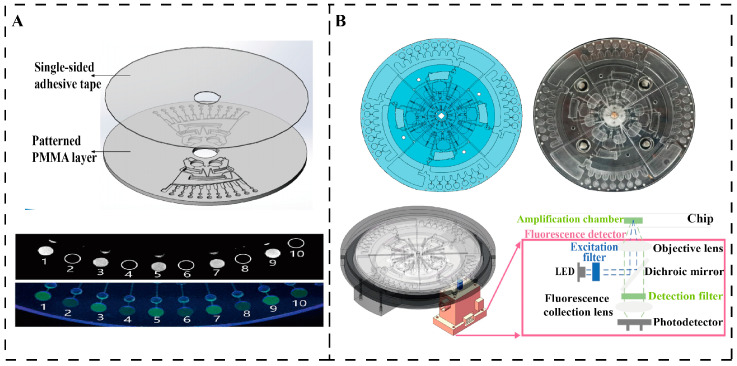
Centrifugal microfluidic detection methods. (**A**) A schematic diagram of a centrifugal chip for bacteria detection and detection results from [82]. Copyright 2017, Springer Nature. (**B**) Centrifugal microfluidic chip performs rotating fluorescence scanning detection by installing a fluorescence detector on the channel [83]. Copyright 2024, MDPI.

Optical inspection is one of the most commonly used methods due to the optically transparent and non-contact nature of the polymer Lab-on-a-Disc (LOAD) [81]. In 2016, Litvinovet et al. developed an innovative platform based on centrifugal sedimentation immunoassays for the rapid detection of bacterial pathogens in water. The principle of the assay is based on the binding of bacteria to antibody-functionalized microbeads, followed by centrifugal sedimentation in density media and quantification of the fluorescence signal to determine the bacterial concentration. The platform simplifies sample handling, detects up to 10 bacteria in just 20 min, and can detect multiple pathogens simultaneously. Compared to the ELISA method, the sensitivity of the assay increased by 10 to 1000-fold, with a detection limit of 10^3^ CFU/mL. Although the system is highly efficient, there is an uneven distribution of low-concentration bacteria on the microbeads, which requires further optimization [7]. The microfluidic bacterial detection chip developed by He et al. was fabricated using polymethylmethacrylate (PMMA) material with dimensions of 60 × 3 mm and integrated lysis, clarification, mixing, and reaction functions such as that shown in Figure 8A. Its lysis unit lyses bacteria by agitation and collision of zirconia beads, followed by centrifugal force to transfer the lysate to a clarification chamber for mixing with the LAMP mother liquor. The chip uses centrifugal force, capillary valves and siphon pipework to control the flow of liquid to ensure no cross-contamination and LAMP amplification. The entire process took only 70 min, and the chip was highly automated and successfully detecting six bacterial species [82]. The centrifuge-based microfluidic chip designed by Lu achieves genome DNA extraction from *E. coli* with an efficiency comparable to that of conventional kits. By actively collecting magnetic beads, the chip reduces time consumption by 60%, significantly enhancing operational efficiency, as shown in Figure 8B. The centrifuge disc precisely controls on-chip fluid at a maximum speed of 1500 rpm, sequentially passing through the pre-loading zone, nucleic acid extraction structure, and LAMP reaction zone, enabling bacterial lysis, nucleic acid extraction and purification, LAMP reaction, and real-time detection. The entire detection process is fully automated and completed within 60 min, with a detection limit of 10^2^ CFU/mL [83].

Researchers continue to explore the LOAD platform for waterborne pathogens detection, and research in this area is showing a growing trend. Advantages of the centrifugal platform include the elimination of the need for pneumatic interfaces and pumps, liquid handling independent of sample properties, full integration, automation, and miniaturization. In the future, centrifugal microfluidics-based aquatic pathogen detection technology will develop toward high-throughput integration, intelligence, and rapid detection in the field. By optimizing the valve system and disc layout, the maximum speed of the device can be reduced to 1500 rpm, which significantly reduces power consumption and improves portability. Combined with smartphone drive and IoT technology, real-time monitoring without an external power supply is possible. Multi-step integration enables testing to be completed in less than 60 min. In addition, the integration of 3D printed kits and microfluidic chips will drive automation, while low-cost materials and large-scale production will further address the needs of resource-limited regions.

### 4.3. Droplet-Based Microfluidics Detection of Waterborne Pathogens

Droplet microfluidics extends the lab-on-a-chip solution to microbial detection through sophisticated on-chip integration design for single-cell encapsulation, rapid droplet generation, and precise manipulation, making it an important tool for microbial detection, especially in the detection and characterization of waterborne pathogens. Droplet microfluidics offers the advantages of high sensitivity, high throughput, and low sample consumption. The detection process includes sample pretreatment, droplet generation, pathogen identification, signal amplification, and analysis. To optimize the technique, the researchers developed a heating reaction, hydrodynamic simulation, and superhydrophobic electromagnetic needle to improve the detection efficiency and manipulation performance.

**Figure 9:**
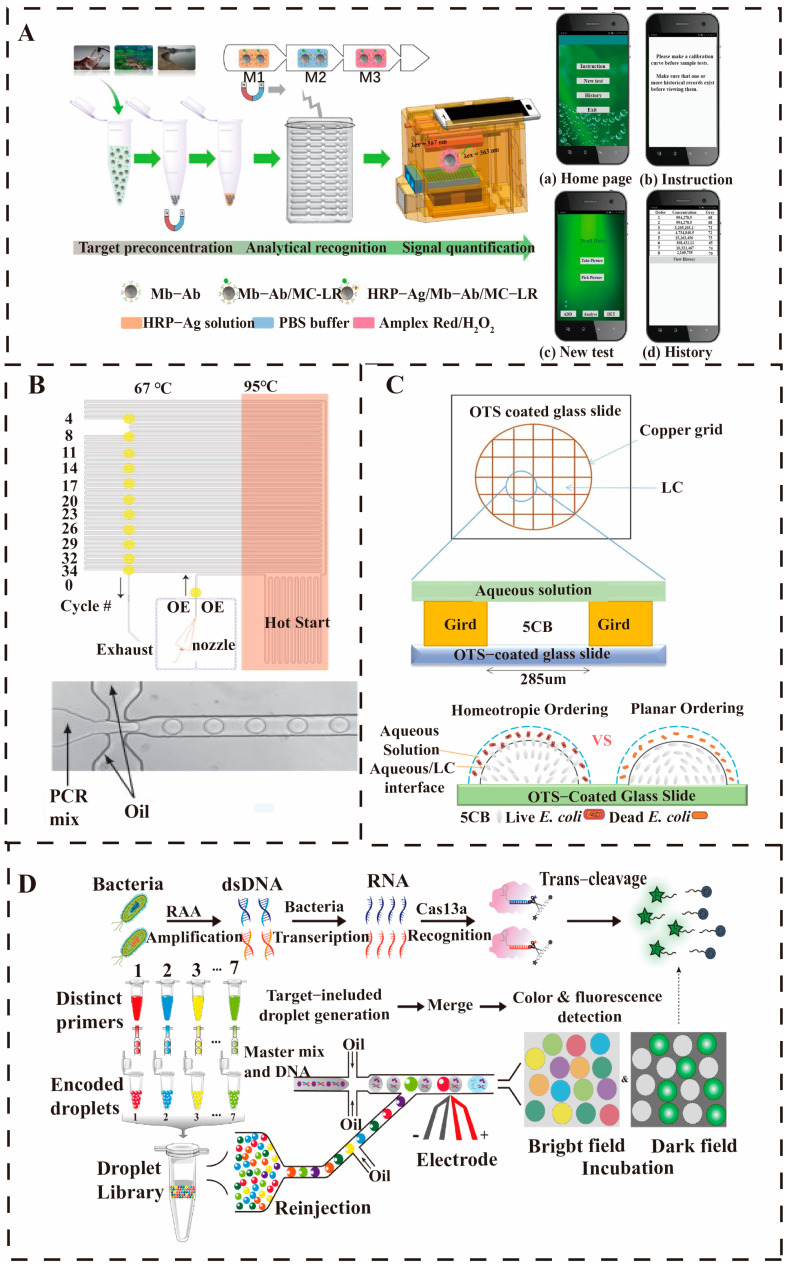
Droplet-based microfluidic detection chip. (**A**) An open-surface droplet microfluidic device for monitoring microcystin-LR in reservoirs and detection principle [86]. Copyright 2020, American Chemical Society. (**B**) Microdroplet-based PCR structure [88]. Copyright 2008, American Chemical Society. (**C**) LC film sensing device, diagram of the ordered state of 5cb droplets at the live and dead *E. coli* water suspension LC interface [89]. Copyright 2018, Elsevier. (**D**) CRISPR/Cas13a-based droplet microfluidics for multiplexed bacterial detection [90]. Copyright 2024, Elsevier.

Open droplet microfluidics combines the phenomenon of electro-wetting with the application of an electric potential to control the movement, merging, splitting, and mixing of droplets, which is characterized by low reagent consumption, parallel operation, and high-throughput stepper motor and microcontroller are programmed to automate the operation of a 10 μL droplet, allowing for complex droplet path movement and rapid mixing. Droplet merging combined with backscattered light detection on a superhydrophobic surface enables latex immunoagglutination assays with a detection limit of 85 CFU/mL for *E. coli*, and the platform can be reused more than 100 times [84]. To further optimize droplet microfluidics, the surface tension-assisted removable open microfluidics (STORM) system uses surface tension to assist droplet movement, significantly reduces energy consumption through surface chemical modifications and mechanical treatments, and eliminates the need for micropumps to direct the flow of liquids, increasing the frequency and efficiency of droplet generation [85]. High-throughput parallel detection excels in this technology, as shown in Figure 9A, with hundreds or thousands of reactions running simultaneously on a single chip, quantification of microcystins in water by open-surface droplet microfluidic magnetic sensors, with a detection limit of 1.2 × 10^−5^ μg/L [86]. Another method combined a giant magnetic impedance (GMI) sensor for ultrasensitive detection and quantification of *E. coli* O157:H7 with a detection limit of 50 CFU/mL [87].

In the early days of closed-droplet microfluidics in the 21st century, researchers have begun to explore the use of droplet microreactors for single-molecule detection and analysis. In 2001, Lagally et al. achieved the first quantification and PCR amplification of single-molecule DNA by droplet microfluidic chips [91]. With the rise of digital PCR technology, droplet microfluidics has become an ideal platform for pathogen nucleic acid molecule detection. In 2008, Margaret et al. achieved the first high-throughput digital PCR experiments using droplet microfluidics, which pushed forward the extension of the technology to single-cell analysis for capturing and analyzing single-cell gene expression and metabolites such as shown in Figure 9B, opening a new pathogen identification and phenotyping [88]. As the technology matures, droplet microfluidics is gradually being applied to the detection of pathogens in real clinical and environmental samples [92,93]. Wei et al. developed a technology based on a liquid crystal (LC) sensing platform, which monitored the chitosan-chitosan (CS-GO) induced phospholipid membrane rupture process of *E. coli* in real time through polarized light microscopy (POM). As shown in Figure 9C, using liquid crystal droplets, the study successfully distinguished between living and dead *E. coli*, showing that CS-GO can destroy the integrity of the cell membrane, and the change in the liquid crystal optical signal reflects the degree of damage to the bacterial membrane [89]. Researchers have integrated a non-amplified assay in a digital microfluidic device for the detection of *Legionella* in water, as well as for the analysis of viruses, bacteria, and parasites in blood, urine, and environmental waters [94,95,96,97]. Shang et al. proposed a high-throughput platform based on droplet microfluidics and clustered regularly interspaced short palindromic repeats associated protein 13a (CRISPR/Cas13a) assay, combined with recombinase-assisted amplification (RAA) and droplet encoding strategy to achieve efficient detection of pathogens. As illustrated in Figure 9D, the simultaneous detection of seven pathogens was achieved through CRISPR/Cas13a signal amplification, droplet confinement effect, and change in droplet color, showing detection performance comparable to quantitative real-time PCR (qPCR). The droplet size and merging ratio can be adjusted according to demand, further expanding its application range, and its high-throughput characteristics make it suitable for the detection of other pathogenic bacteria [90]. Although there are currently problems, such as the inability to achieve absolute quantification and dependence on external equipment, future work will focus on solving these limitations to promote its widespread application in clinical, environmental, and food safety.

To enhance the efficiency and applicability of droplet microfluidics in bacterial detection, researchers have introduced several innovative optimization strategies. Suo et al. developed a thermosetting oil-based pico-droplet array capable of isolating bacterial cultures at the cuticle level. By employing fluorescent signal conversion for digital quantification, this approach achieved sensitive detection of *E. coli* at concentrations as low as 500 CFU/mL with in 4 h, detection limit of 1 CFU/droplet [94]. In another advancement, Li et al. designed a double-layer droplet continuous-flow PCR (CF-PCR) microfluidic chip that significantly improves amplification efficiency through optimized fluidic pressure and droplet fusion. This system enabled rapid detection of *P. gingivalis*, generating a positive signal in just 12 min while simultaneously overcoming the gas permeation limitations of polydimethylsiloxane (PDMS). These developments highlight the potential of droplet microfluidics for high-throughput, rapid, and sensitive bacterial diagnostics [95].

Researchers have also continued to expand the application of droplet microfluidics in pathogen detection, including viability analysis, resistance detection, targeted enrichment, and high-throughput screening, further unlocking the potential of the technology. In conclusion, closed-droplet microfluidics has evolved from proof-of-concept to mature and industrialized technology in waterborne pathogens detection, providing a high-throughput, high-sensitivity, and contamination-free detection mode. By integrating 3D printing technology and novel materials, the manufacturing cost can be significantly reduced, and the stability of the device can be improved. Deep learning-driven image analysis will enable real-time classification of single-cell pathogens within droplets, with an accuracy rate expected to exceed 90%. A multifunctional droplet platform incorporating the CRISPR-Cas system will enable simultaneous detection of nucleic acid and protein markers, while a portable design and IoT technology will drive on-site monitoring applications. The technology is ultimately expected to form a closed-loop system from sample preprocessing to result analysis, providing innovative solutions for global water safety monitoring.

### 4.4. Paper-Based Microfluidics Detection of Waterborne Pathogens

The paper-based microfluidic analysis devices (μPAD), an emerging microfluidics technology platform that combines traditional paper materials with microfluidics technology, is attracting attention as a promising rapid on-site diagnostic tool for rapid, portable, and low-cost biomolecular analyses and has emerged as a cost-effective, efficient, and highly sensitive method for pathogen detection and analysis [98].

**Figure 10:**
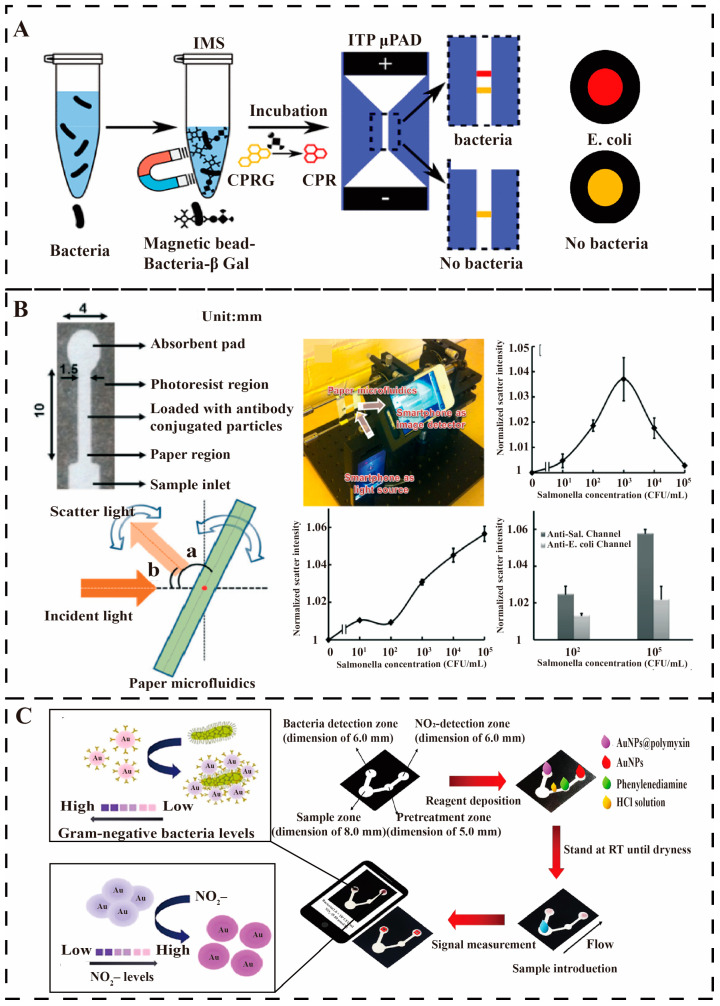
Paper-based microfluidics for pathogen detection. (**A**) The paper-based separation device detects *E. coli*, and two visible color bands display the detection results in [33]. Copyright 2019, American Chemical Society. (**B**) Smartphone detection of *Salmonella* and cross-reaction with *E. coli* resistance and detection results from, the angle of incident light to paper (angle a) and the angle of light scatter from paper (angle b). [99]. Copyright 2013, Royal Society of Chemistry. (**C**) *E. coli* was detected using a μPAD and a smartphone reader [100]. Copyright 2023, American Chemical Society.

At the beginning of the 21st century, based on traditional microfluidic technology, researchers began to experiment with the use of cheap and readily available paper as a microfluidics substrate, and in 2009, Robert reported the first concept of a paper-based microfluidics chip in the journal Trends in Analytical Chemistry [101]. Paper-based microfluidics structures show explosive growth in 2020 as researchers prove the feasibility of using paper-based microfluidics chips for pathogen detection [102]. Key breakthroughs in the preparation process and detection strategy of paper-based microfluidics chips, such as the use of etching technology to accurately prepare three-dimensional channel structures and the use of precious metal nanoparticles to achieve high-sensitivity signal amplification and detection, lay the technological foundation for the multi-detection of pathogens [103,104].

Continuing advances in materials and processing technologies have enabled researchers to increase the use of paper-based microfluidic chips for real-world waterborne pathogens monitoring. Federico et al. present a new method for the rapid detection of very low concentrations of bacteria that can significantly increase sensitivity through two preconcentration steps (Figure 10A). First, samples are mixed with antibody-modified magnetic particles and β-galactosidase-conjugated free antibodies, and the target bacteria are concentrated by immunomagnetic separation. Next, these bacteria are reacted with chlorophenol red-β-D-galactoside (CPRG) to produce chlorophenol red (CPR), which is used to detect the bacterial concentration. The second step utilizes a paper-based analytical device to separate and focus CPR and CPRG, significantly improving the detection limit and enhancing the robustness of the assay by forming test and control bands. The method successfully detected 9.2 CFU/mL of *E. coli* within 90 min [33].

Since 2013, paper-based microfluidic chips have been developed in the direction of integration with mobile terminals such as smartphones to achieve automated operation, intelligent analysis, wireless data transmission, and to improve the intelligence of on-site testing. As shown in Figure 10B, Park et al. designed a paper-based microfluidic channel pre-loaded with *Salmonella* antibodies and *E. coli* antibodies. When the paper-based microfluidic device is immersed in a *Salmonella* solution, the antibody-bound particles bind to the paper fibers, and an immunoagglutination reaction occurs. The bacterial concentration is determined by evaluating the Mie scattering of digital images taken by a smartphone at an optimal angle and distance and quantifying the degree of immune agglutination through a processing program [99]. Somvanshi et al. have improved paper-based microfluidics by combining it with a multiplexed sensing platform for the simultaneous detection of *E. coli* O157:H7 and *Salmonella typhimurium*. Color signals were read and quantified using image analysis to measure bacterial concentrations and quantitative detection was achieved by a paper-based microfluidic device. Color results show a linear relationship between *E. coli* O157:H7 and *Salmonella typhimurium* over a wide concentration range (10^2^ CFU/mL to 10^8^ CFU/mL) with limits of detection of 10^3^ CFU/mL and 10^2^ CFU/mL, respectively [105].

Alternatively, Zhao et al. developed a waxed paper enzyme-linked immunosorbent assay (P-ELISA) with a total operating time of fewer than 3 h, requiring only 5 μL of the sample. The limit of detection for *E. coli* O157:H7 reached 10^4^ CFU/mL, which is an order of magnitude higher than that of conventional ELISA (C-ELISA) [24]. Subsequently, this paper-based microfluidics was combined with recombinase polymerase amplification (RPA) for the detection of *Salmonella* typhimurium. Design of single-stranded DNA ‘pull-down’ SERS nanoprobes. The detection limit for *Salmonella typhimurium* was approximately 3–4 CFU/mL, and the dynamic detection range was extended to 1–10^8^ CFU/mL with a reduced detection time of less than 45 min. This work further extends the range of CRISPR-based diagnostics [106]. Kawin et al. monitored *E. coli* in water using polymyxin-functionalized gold nanoparticles (AuNPs) that undergo a color change due to aggregation. Using a smartphone app, the detection limit for *E. coli* was determined to be 2.0 × 10^2^ CFU/mL by measuring the color change caused by aggregation and anti-aggregation reactions, as shown in Figure 10C. The assay is highly selective, independent of Gram-positive bacteria, and achieves acceptable recoveries, extended stability, reduced costs, and ensures long-term viability [100].

Paper-based microfluidics analysis devices (μPADs) represent a transformative approach for aquatic pathogen detection, combining paper substrates with microfluidics to create rapid, portable, and cost-effective diagnostic platforms. Future developments will focus on enhancing functionality through optimized wax printing and conductive ink technologies for rapid prototyping and reduced manufacturing costs. Integration with smartphone-based image analysis and IoT systems will improve real-time quantitative detection capabilities, achieving picomolar-level sensitivity. Advanced designs incorporating multi-electrode arrays will enable simultaneous multi-target detection for complex samples, while graphene oxide coatings will boost sensitivity through increased surface area and color uniformity. Although challenges remain in standardizing large-scale production and clinical translation, μPADs are poised to become indispensable tools for water safety monitoring, particularly in resource-limited settings, offering intelligent, on-site diagnostic solutions with expanding applications in portable environmental monitoring.

## 5. Conclusions and Outlook

Portable microfluidics systems play an important role in risk assessment, epidemic prevention, and vaccine distribution in resource-poor environments, especially for monitoring waterborne pathogens. Portable microfluidic systems have become an important tool for on-site detection of waterborne pathogens (bacteria, viruses, and parasites) due to their miniaturization, integration, and automation features. Compared with traditional laboratory methods, microfluidic chips can complete sample preprocessing, nucleic acid amplification, and signal detection within tens of minutes, significantly improving detection efficiency. For example, isothermal amplification (LAMP)-based microfluidic chips have achieved single-copy sensitivity, making them suitable for screening low concentrations of pathogens [107,108]. In addition, by combining nanomaterials (gold nanoparticles) and biosensors, some of the systems can detect multiple pathogens at the same time, meeting the needs of complex water environment monitoring [22,100]. Solutions combining biosensors and nucleic acid amplification (NAA) technology also show potential in improving detection efficiency and deserve further research and development [5].

Based on the contents of the review, Table 1 summarizes the key parameters of microfluidics in separation and detection methods, including the separation mechanism, detection method, separation efficiency, detection performance, and other indicators.

Microfluidics holds significant promise for rapid detection, yet its practical deployment is hindered by several challenges, including sample impurities that clog channels or inhibit reactions, necessitating integrated pretreatment modules like centrifugation or filtration, as well as temperature fluctuations that disrupt nucleic acid amplification, requiring built-in temperature control systems for stability. Additionally, reliance on external power supplies and specialized operators limits long-term monitoring in remote areas, driving the need for improvements in anti-interference capability, operational simplicity, and energy efficiency. To address these limitations, researchers are pursuing multi-technology integration, such as combining CRISPR-Cas systems with microfluidics to enhance specificity, leveraging IoT for real-time data upload and remote monitoring, and adopting acoustic-fluid-driven hybrid technologies or micropump-valve systems to automate and refine fluid control [106,109]. Future advancements may involve 3D printing and degradable materials to lower chip costs and enable mass production, while machine learning algorithms could optimize multi-pathogen identification, minimize false positives/negatives, and bolster testing reliability.

As technology matures, portable microfluidic systems will play a greater role in public health, environmental monitoring, and disaster response. For example, long-term deployment in drinking water sources or sewage treatment plants can dynamically track trends in the spread of antibiotic resistance genes (ARGs) [110]. In resource-poor areas, low-cost, easy-to-operate testing equipment will help with early outbreak warning. In addition, standardized production and policy support will accelerate the spread of the technology, making it a core tool for global water safety monitoring and providing critical support for responding to pollution emergencies and infectious disease prevention and control.

Overall, the development of microfluidic technology has improved the sensitivity, speed, and specificity of pathogen detection and reduced equipment costs and energy consumption through technologies such as nanomaterial platforms, droplet microfluidics, and chip lab systems. Future devices may combine machine learning and IoT technologies to further optimize multiple detection functions, adapt to the needs of field applications, and promote widespread deployment in public health and environmental monitoring.

## Figures and Tables

**Table 1:** Overview of microfluidics-based isolation and detection of waterborne pathogens.

Separation Method	Detection Method	Bacteria	Separation Efficiency	Detection Limit	Reference
Staggered Flow Microfiltration Membrane	Microscopic Imaging	*E. coli*	90%	/	[48]
Periodic Fluid Drive + Membrane Filtration	Fluorescence Microscopy	*E. coli*	100 times	10^2^ CFU/mL	[49]
Membrane	Electrochemical Sensing	*Salmonella*	95%	10^2^ CFU/mL	[50]
3D Printed Bionic Membrane	Optical Microscopy	*E. coli*	85%	/	[51]
Photofluidics	High-Speed Microscopy	*E. coli*	90%	10^3^ CFU/mL	[5]
DMF combined with optical tweezers	Fluorescence Microscopy	*E. coli*	70%	10 CFU/mL	[57]
Infrared Optical Tweezers	Fluorescent Labeling	*Saccharomyces cerevisiae*	95%	Single Bacteria	[58]
Miniaturized Fiber Optic Tweezers	Quantum Dot Labeling	*E. coli*	85%	Single Bacteria	[59]
Hydrodynamic Focusing	Fiber Optic Transmission Spectroscopy	*E. coli*	90%	10^2^ CFU/mL	[54]
DEP	Fluorescence Microscopy	*Lactobacillus*	96%	Single Bacteria	[61]
ICP	Smartphone Fluorescence Imaging	*E. coli*	/	10^2^ CFU/mL	[63]
Inertial Microfluidics	High-speed microimaging	*Saccharomyces cerevisiae*	95%	/	[71]
Inertial Microfluidics	Fluorescent Antibody Labeling	*Giardia duodenalis*	78%	1 cyst/g	[73]
Inertial Microfluidics	Fluorescent labeling	*Jakobid flagellates*	90%	/	[70]
DLD	Electrochemical Impedance Sensing	*E. coli*	92%	10 CFU/mL	[74]
SAW	qPCR	*E. coli*	88%	10^2^ CFU/mL	[69]
/	Flow Cytometry	*E. coli*	/	10^3^ CFU/mL	[76]
Microporous Array Aptamer-Ag10NPs	Brightfield imaging analysis	*Klebsiella pneumoniae*	85%	10^3^ CFU/mL	[78]
SERS	SERS Spectroscopy	*Staphylococcus aureus*	80%	Single Bacteria	[79]
Chemiluminescent Digital Microtiter Array	High-Sensitivity CCD Imaging	*E. coli*	95%	1 CFU/mL	[80]
Centrifugal sorting	LAMP Amplification	*Listeria monocytogenes*	>90%	10^2^ CFU/mL	[82]
Electromagnetic Field Assisted Magnetic Beads	Real-time fluorescence LAMP	*E. coli*	/	10^2^ CFU/mL	[83]
Antibody-Modified Magnetic Beads	Fluorescence Microscopy	*Shigella*	/	10^3^ CFU/mL	[7]
/	Multiplex PCR	*E. coli*, *Listeria monocytogenes*, *Salmonella typhimurium*	/	10^2^ CFU/mL	[84]
/	Fluorescent Labeling	*Saccharomyces cerevisiae*	/	/	[85]
/	GMI	*E. coli O157:H7*	85%	10 CFU/mL	[87]
Droplet Encapsulation	CRISPR/Cas13a	*Staphylococcus aureus*	/	10 CFU/mL	[90]
Aerosol particles	Flow-through Fluorescence	*Bacillus*	/	1 CFU/mL	[92]
Centrifugation	Smartphone Camera	*Salmonella*	85%	10^3^ CFU/mL	[99]
CRISPR-Cas12a targeted shearing	SERS	*Staphylococcus aureus*	/	1 CFU/mL	[106]
